# Efficacy of non-operative treatment of patients with knee arthrofibrosis using high-intensity home mechanical therapy: a retrospective review of 11,000+ patients

**DOI:** 10.1186/s13018-022-03227-w

**Published:** 2022-07-06

**Authors:** Shaun K. Stinton, Samantha J. Beckley, Thomas P. Branch

**Affiliations:** 1 ArthroResearch LLC, 441 Armour Place NE, Atlanta, GA USA; 2 Ermi LLC, 2872 Woodcock Blvd. Suite 100, Atlanta, GA USA

**Keywords:** Arthrofibrosis, Knee stiffness, Mechanical therapy, Non-operative treatment, High-intensity

## Abstract

**Background:**

Recovery from knee surgery or injury can be hindered by knee arthrofibrosis, which can lead to motion limitations, pain and delayed recovery. Surgery or prolonged physical therapy are often treatment options for arthrofibrosis, but they can result in increased costs and decreased quality of life. A treatment option that can regain lost motion without surgery would help minimize risks and costs for the patient. The purpose of this study was to determine treatment efficacy of high-intensity home mechanical stretch therapy in patients with knee arthrofibrosis.

**Methods:**

Records were reviewed for 11,000+ patients who were prescribed a high-intensity stretch device to regain knee flexion. Initial and last recorded knee flexion and days between measurements were available for 9842 patients (Dataset 1). Dataset 2 was a subset of 966 patients from Dataset 1. These 966 patients had separate more rigorous measurements available from physical therapy notes (Dataset 3) in addition to data from the internal database (Dataset 2). Within and between dataset statistics were calculated using *t* tests for comparison of means and Cohen’s d for determination of effect size.

**Results:**

All dataset showed significant gains in flexion (*p* < 0.01). Mean initial flexion, last recorded flexion and flexion gain were 79.5°, 108.4°, and 29.9°, respectively in Dataset 1. Differences between Datasets 2 and 3 had small effect sizes (Cohen’s *d* < 0.17). The were no significant differences when comparing workers’ compensation and non-workers’ compensation patients. The average last recorded flexion for all datasets was above the level required to perform activities of daily living. Motion gains were recorded in under 60 days from device delivery.

**Conclusions:**

High-intensity home mechanical stretch therapy was effective in restoring knee flexion, generally in 2 months or less, and in avoiding additional surgery in severe motion loss patients regardless of sex, age, or workers’ compensation status. We believe high-intensity stretching should be considered in any patient who is at risk for a secondary motion loss surgery, because in over 90% of these patients, the complications and costs associated with surgery can be avoided.

## Background

Approximately 2 million patients undergo total knee arthroplasty or arthroscopic knee surgery each year in the United States [1, 2]. The majority of these patients achieve a good clinical outcome including a return to functional range of motion (ROM) and pain-free activities of daily living within 2 years of their surgery. However, knee arthrofibrosis characterized by stiffness in the joint associated with motion loss can occur in some patients after knee surgery or injury. There are an estimated 85,000 patients who develop postoperative knee arthrofibrosis in the United States per year [3]. The types of surgeries and the percentage of patients that have continued severe motion loss beyond 1 year after that surgery (in parentheses) include: anterior cruciate ligament reconstruction (2–35%) [4], multiple ligament reconstruction (13–22.4%) [5], total knee arthroplasty (1.3–12%) [6], tibial plateau fracture repair (14.5%) [7], and femur fracture repair (29.5%) [8]. A standard protocol of 6–8 weeks of physical therapy is effective in treating the majority of patients with arthrofibrosis [9]; however, outlier patients (those whose motion recovery does not respond to traditional physical therapy) can be left with marked loss of function and significantly increased healthcare costs due to arthrofibrosis. Patients with unresolved arthrofibrosis in the knee can develop severe, disabling pain and motion limitations that can interfere with activities of daily living [10]. These conditions significantly delay recovery which, in turn, delays return to work or sport.

When a patient’s motion loss recovery fails to respond to traditional physical therapy, a critical clinical pathway decision is reached. Currently, this decision is between a secondary surgery to manage excessive scar tissue formation/motion loss or continued efforts to recover motion with traditional physical therapy. Many surgeons resort to the risk of secondary surgery such as a manipulation under anesthesia (MUA) with or without an arthroscopic lysis of adhesions which restarts the timeline of recovery and results in increased direct costs (secondary surgery, additional rehabilitation, and medication) and indirect costs (missed work) while reducing quality of life for the patient. In the United States, an MUA is required in up to 7.3% of total knee arthroplasty patients [11–19] and 11.3% of arthroscopic knee patients [20, 21].

A third option for recovery of motion loss due to arthrofibrosis is high-intensity home mechanical stretch (HIS) therapy which can help avoid prolonged physical therapy or secondary surgery. The HIS device described in this study is hydraulically driven and is used in a patient’s home allowing the patient to apply a stretch to their knee multiple times per day up to a level of force that a physical therapist can apply in a clinical setting [22]. This high-intensity stretch is completely patient controlled with the stretch focused at the end-range of motion. Therefore, this device focuses on maximizing total end range time (TERT), a product of intensity, frequency and duration of passive stretching, therefore, encouraging plastic deformation of the soft tissue [23]. The goal of HIS therapy is to achieve lasting gains in knee motion by permanently elongating scar tissue through load deformation.

The HIS device described in this study has been previously shown to have a success rate of over 90% in both preventing a secondary surgery and in regaining functional ROM for severe motion loss patients [24, 25]. The success rates associated with treatment using an HIS device show that patients with a severe flexion deficit have a high likelihood of regaining functional ROM. No complications have been reported using the HIS device, and therefore, this choice of treatment avoids the additional costs and/or risks that are associated with motion loss surgery or prolonged physical therapy. While the earlier studies are promising, they are small in scale and data from a larger patient population are needed to validate the clinical benefit and potential cost savings from using HIS therapy.

Therefore, the purpose of this retrospective study was to analyze data from more than 11,000 patients who utilized an HIS device to recover their knee flexion loss with the intent to determine treatment efficacy. The primary hypothesis of the study was that patients treated with HIS therapy would achieve significant gains in knee flexion. Secondary hypotheses were that the motion gains would be sufficient for patients to avoid a secondary surgery and to perform activities of daily living.

## Methods

### Data collection

**Fig. 1:**
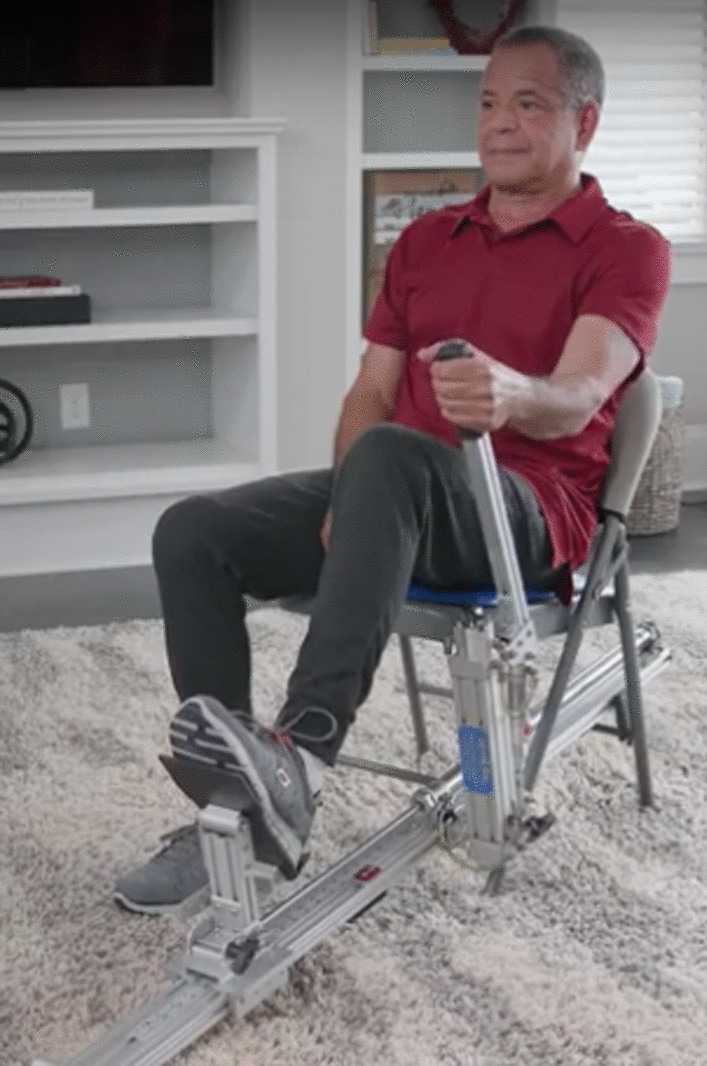
Ermi Knee Flexionater

**Fig. 2:**
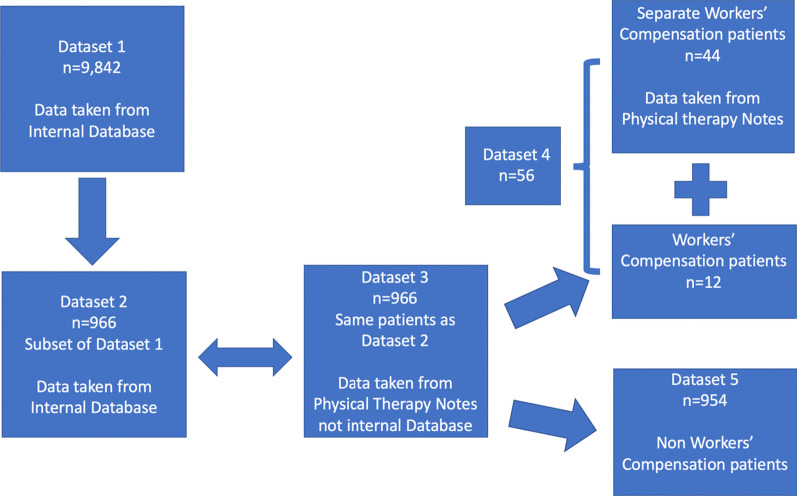
Flowchart of datasets used for analysis

This retrospective study was determined to be exempt from IRB review and a waiver of authorization was granted. Records from more than 11,000 patients who were prescribed an HIS device (the Ermi Knee Flexionater—Fig. 1) for motion recovery between 2008 and 2018 were reviewed. Patients who never received a device due to an insurance denial or that opted out of treatment were removed along with duplicate patients which left 9,842 patients for analysis (Dataset 1, as shown in Fig. 2). Documents in the internal database included clinical notes from doctors and physical therapists and other related documents, such as letters of medical necessity, intake paperwork, recertification paperwork, etc. Range of motion data were logged into the internal database during the process of treating the patients. In some cases, there was not a record of the exact date of each measurement and it was not known whether all measurements were taken with a goniometer.

The variables of interest were: (1) the knee flexion measurement taken closest to the time the device was delivered to the patient; (2) the last recorded knee flexion measurement; (3) the number of days between those measurements; and (4) the number of days between device delivery and the last recorded knee flexion measurement. These variables were extracted from each patient’s records in the internal database.

### Device protocol

Patients were prescribed an HIS device after reaching a plateau in their motion recovery after at least 4 weeks of treatment with a standard protocol of physical therapy. These patients were consistently unable to meet their individual knee flexion goals that were set by the patient and surgeon based on the injury type, surgical procedure, contralateral knee flexion, age and sex of the patient, and pre-operative knee flexion. Patients used an HIS device to assist flexion stretching of their knee during three treatment sessions per day. During each session, the patient advanced the stretch to the maximum tolerable flexion, maintained the stretch for 10 min, and then released the stretch for 10 min. This was followed by another identical 10 min period of end-range stretch. Patients were instructed to stretch the joint to a level of discomfort just below the pain threshold. The device included a hydraulic mechanism that allowed the patient to control the force of the stretch at variable loads and sensitivity up to a level equal to what is applied by a physical therapist [22]. The patient’s own active involvement in stretching using the device and the precise control provided by the hydraulic mechanism allowed the patient to establish a kinesthetic sense and feedback that is unique when compared with other forms of mechanical therapy. The patient could easily change the intensity of the stretch during the treatment period by pumping the device lever or using a quick-release mechanism.

### Subset datasets

A subset of 966 patients taken from the larger Dataset 1 was defined as Dataset 2 (Fig. 2). This dataset only included the data from 966 out of 9842 patients, but the data for these 966 patients was the same between Dataset 1 and Dataset 2. These 966 patients were selected for the subset, because in addition to the records from the internal database, these patients also had more rigorous ROM data that were available from physical therapy notes. These collected measurements were taken in various clinics by a physical therapist using a goniometer and included the date of the measurement. These physical therapy ROM measurements for the 966 patients were defined as Dataset 3. The data were from patients that received the HIS device between 2010 and 2013. Data were aggregated from detailed reports that were previously prepared for physicians from select orthopedic clinics in Illinois, Texas and Florida or for payors including Independence Blue Cross Blue Shield (BCBS) in Pennsylvania, BCBS of Illinois, BCBS of Florida, and BCBS of North Carolina.

The initial recorded knee flexion was the measurement that was taken closest to the time of delivery of the HIS device. Further measurements were taken when a recertification of medical necessity was required, which was generally on a monthly basis. The last recorded knee flexion was the most recent measurement available for each patient. Since the last recorded clinical measurement would be from the beginning of the last recertification period, the measurement is most likely lower than the patient’s final ROM after completing treatment with the HIS device and could be significantly lower in some patients. In each patient, the initial and last recorded measurements were required to be from the same source for consistency (i.e., the same physical therapy clinic). This allowed for more consistent reporting of ROM gains from patients treated at different locations.

Passive ROM measurements (PROM) were preferred as more patients had PROM measurements available in their clinical notes and the measurements in the internal database were PROM; however, in a small percentage of patients (< 5%), only a complete set of active ROM measurements (AROM) was available. In several instances, there were datasets with only one PROM and one AROM available. In such cases, the data was only used for analysis if the initial knee flexion measurement was a PROM measurement with an AROM measurement available for the second ROM and not vice versa. Since PROM is generally larger than AROM, in cases with PROM for the first measurement and AROM for the second measurement, the patient’s improvement in ROM would most likely be underestimated, and not overestimated.

Exclusion criteria included: (1) Patients who did not have 2 sets of knee flexion measurements; (2) Patients who did not have an initial measurement taken within 30 days of device delivery; (3) Patients whose second measurement was not at least 10 days post-device delivery; and (4) Patients who did not have at least 14 days between measurements.

Since the subset of 966 patients had ROM data collected from two sources (internal database and physical therapy notes), these patients could be used to validate the findings from the 9842 patients in Dataset 1 to see if the data collection methods for Dataset 1 were comparable to the more rigorous data in Dataset 3 collected from physical therapy notes. Statistical comparisons were performed between the two datasets for the 966 patient subset (Dataset 2 and Dataset 3). Within dataset and between dataset statistics were calculated using *t* tests for comparison of means and Cohen’s d for determination of the effect size.

## Results

### Range of motion data

Range of motion data for the 9842 patients in Dataset 1 are shown in Table 1. The last recorded flexion was significantly greater than the initial flexion (*p* < 0.01). Range of motion data for the subset of 966 patients taken from the internal database (Dataset 2) and ROM data from physical therapy notes (Dataset 3) are shown in Table 2. The last recorded flexion was significantly greater than the initial flexion for both data sources (*p* < 0.01).

**Table 1:** Data for the 9842 patients in dataset 1. SD = standard deviation; CI = confidence interval

	Mean ± SD	Median	95% CI
Flexion at device delivery (°)	79.5 ± 20.0	82	79.1–79.9
Last recorded flexion (°)	108.4 ± 15.3	110	108.1–108.7
Flexion gain (°)	28.9 ± 20.0	25	28.5–29.3

**Table 2:** Range of motion data for the 966 subset patients who had data available from the internal database (Dataset 2) and from physical therapy notes (Dataset 3). Data are presented as mean ± standard deviation

	Dataset 2 (Internal Database)	Dataset 3 (Physical Therapy Notes)
*n*	966	966
Mean flexion at device delivery (°)	80.7 ± 19.8	85.3 ± 19.5
Mean last recorded flexion (°)	109.8 ± 14.7	110.7 ± 14.7
Mean flexion gain (°)	29.2 ± 19.7	25.5 ± 18.5
Mean days between delivery and last recorded flexion (days)	NA	45.1 ± 37.5
Median flexion at device delivery (°)	85	90
Median last recorded flexion (°)	110	112
Median flexion gain (°)	25	22
Median days between delivery and last recorded flexion (days)	NA	43

When Datasets 2 and 3 were compared using *t* tests, initial flexion, last recorded flexion, and gain in flexion were all significantly different. However, due to the large sample sizes, relatively small differences were statistically significant. The largest difference between the groups was in the initial range of motion (4.6° difference) but this only had a small effect size (Cohen’s *d* = 0.16). The average initial flexion in both Dataset 2 and Dataset 3 was below the expected clinical level that would likely require a motion restoring surgery [26]. The stricter inclusion criteria for the physical therapy data in Dataset 3 may contribute to this difference, because some of the initial measurements in the data from the internal database (Dataset 2) could have been taken a month or more prior to device delivery. The patient may have been able to gain some knee flexion in physical therapy in that month which would not be reflected if there was no documented measurement. The last recorded flexion and the gain in flexion were similar between Datasets 2 and 3 with small differences of 0.9° and 3.7° degrees, respectively. Both last recorded flexion and flexion gains had small effect sizes as calculated via Cohen’s d (0.04 and − 0.14, respectively).

Table 3 shows treatment efficacy based on the initial range of motion of the patient. A lower starting range of motion results in a greater ROM gain on average and a higher starting ROM corresponds to a higher last recorded ROM. In patients who started with ≤ 60° of knee flexion, 76.2% reached at least 90 degrees of flexion. These patients are catastrophic patients who would otherwise undergo additional surgery and struggle to regain the ability to perform activities of daily living.

**Table 3:** Comparison of treatment efficacy by starting range of motion in the large dataset taken from the internal database (Dataset 1). Data are presented as mean ± standard deviation

	≤ 60°	61–75°	76–90°	91–105°	≥ 106°
*n*	1787	1935	3497	1993	630
Flexion at device delivery (°)	47.1 ± 12.5	69.7 ± 3.9	85.0 ± 4.4	97.7 ± 3.9	112.8 ± 5.2
Last recorded flexion (°)	100.6 ± 20.4	103.6 ± 15.0	109.3 ± 12.4	114.1 ± 10.0	122.3 ± 8.5
Flexion gain (°)	53.5 ± 23.1	33.9 ± 15.4	24.2 ± 12.4	16.3 ± 9.9	9.6 ± 7.4

### Treatment time

For the 9842 patients in Dataset 1, there were 8259 patients who had a date for both delivery of the HIS device and the end of device use. The time between device delivery and end of use averaged 75.4 days. The vast majority (91.9%) of these patients used the device for a period of 4 months or less (3.8% treated for < 30 days, 16.6% treated for 30–59 days, 43.1% treated for 60–89 days, and 28.4% treated for 90–119 days).

Specific dates for when the initial flexion measurement and last recorded flexion measurement were taken were not available for Dataset 1. After the initial measurement was taken, there was a flexion measurement taken for each month the patient used the device. The 9842 patients in Dataset 1 required two recertifications on average. The standard protocol is to obtain a ROM measurement 8–12 days prior to the start of the next month of device use. This would mean the second recertification would occur approximately 50 days after device delivery. Although it cannot be calculated, it is likely the time between measurements in the larger dataset was between 50 and 60 days.

For the subset data for the 966 patients taken from physical therapy notes (Dataset 3), the time between the first and last flexion measurement averaged 55.9 days and the time between device delivery and the last recorded measurement averaged 45.1 days. This means the initial measurement was taken an average of 10.8 days prior to device delivery. These time frames are in line with the estimated times for the larger Dataset 1 as described above.

The effect of the initial ROM on the days of use for the subset of patients in Dataset 3 is shown in Table 4. A lower initial ROM leads to longer use, but the largest difference between the groups is less than 10 days.

**Table 4:** Comparison of treatment efficacy by starting range of motion in the physical therapy data for the subset patients (Dataset 3). Data are presented as mean ± standard deviation

	≤ 60°	61–75°	76–90°	91–105°	≥ 106°
n	114	152	330	247	123
Initial flexion (°)	47.6 ± 10.9	69.6 ± 4.1	85.2 ± 4.5	98.1 ± 4.0	113.4 ± 5.2
Last recorded flexion (°)	102.8 ± 20.7	102.3 ± 14.7	110.0 ± 12.1	113.9 ± 10.1	124.2 ± 9.8
Flexion gain (°)	55.2 ± 22.5	32.7 ± 14.4	24.7 ± 12.5	15.8 ± 9.9	10.8 ± 8.2
Days between delivery and last recorded flexion (days)	50.0 ± 41.0	46.3 ± 27.1	48.2 ± 27.5	44.7 ± 34.6	40.6 ± 28.7

### Demographic comparisons

A comparison of ROM data between sexes is shown in Table 5. When comparing males and females, there was not a statistically significant difference in initial flexion, flexion gain, or days of use. There was a statistically significant difference in the last recorded flexion with 2.0° higher flexion in males. All effect sizes were small as measured by Cohen’s d: initial flexion = 0.01; last recorded flexion = 0.10; flexion gain = 0.06; days of use = 0.09.

**Table 5:** Subset demographics taken from physical therapy notes (Dataset 3): 966 total patients; average age 51 years. Data are presented as mean ± standard deviation

	Male	Female	*p* value
*n*	398	568	
Initial flexion (°)	85.4 ± 19.8	85.1 ± 19.3	0.81
Last recorded flexion (°)	111.9 ± 14.0	109.9 ± 15.1	0.04
Flexion gain (°)	26.5 ± 19.9	24.8 ± 17.5	0.17
Days between delivery and last recorded flexion (days)	48.0 ± 32.4	43.2 ± 40.5	0.05

A comparison of ROM data by age group is shown in Table 6. All age groups achieved a last recorded knee flexion that would allow them to complete most activities of daily living [27, 28]. These gains were achieved in 2 months or less on average in all groups.

**Table 6:** Range of motion data from physical therapy notes (Dataset 3) for the 966 patients separated by age

	Age						
	≤ 20	21–30	31–40	41–50	51–60	61–70	≥ 71
*n*	45	43	78	157	398	241	4
Initial flexion (°)	82.3	81.0	79.6	83.4	86.1	88.0	85.0
Last recorded flexion (°)	116.8	119.4	112.6	109.6	109.2	110.4	117.5
Flexion gain (°)	34.6	38.4	33.0	26.2	23.1	22.4	32.5
Days between delivery and last recorded flexion (days)	40.9	49.2	50.7	49.1	46.1	43.9	34.0

Another interesting finding in this study can be seen in the comparison between workers’ compensation patients and non-workers’ compensation patients. In the subset of 966 patients, there were 12 workers’ compensation patients. Data from physical therapy notes was available for 44 other workers’ compensation patients from the same time period (patients that were not in the larger dataset, because they did not have data in the internal database). Those two sets of data were combined to create Dataset 4 (Fig. 2). Dataset 5 was made up of the 954 patients from Dataset 3 that were not workers’ compensation patients. There were no significant differences between Datasets 4 and 5 (Table 7). There were also no significant differences between the 12 workers’ compensation patients and the 954 non-workers’ compensation patients in Dataset 3.

**Table 7:** Comparison of data between workers’ compensation patients and non-workers’ compensation patients. Data are presented as mean ± standard deviation

	Workers’ Compensation	Non-workers’ Compensation	*p* value
*n*	56	954	
Initial flexion (°)	85.4 ± 22.2	85.3 ± 19.4	0.98
Last recorded flexion (°)	109.8 ± 15.1	110.8 ± 14.7	0.63
Flexion gain (°)	24.4 ± 20.7	25.5 ± 18.4	0.68
Days between delivery and last recorded flexion (days)	47.5 ± 33.5	45.1 ± 37.6	0.64

## Discussion

The most important finding in this study is that regardless of sex or age, patients with severe motion loss who were treated with high-intensity home mechanical stretch therapy achieved excellent gains in their range of motion (> 25° on average). These gains were achieved over a relatively short period of time (6–10 weeks). As described by Keating et al., at least 90° of knee flexion is required to perform basic daily activities and the goal of an MUA after knee arthroplasty is to increase flexion in patients who have failed to reach 90° of flexion postoperatively [26]. Patients in the current study achieved an average last recorded knee flexion that was well above the 90° that would typically be an indication for additional surgical intervention. This indicates that treatment using high-intensity home mechanical stretch therapy helped most patients avoid additional surgery and prolonged physical therapy.

The patients treated in this study were on a clinical pathway to be considered for surgical intervention with an MUA and/or lysis of adhesions. The results of this study provide further evidence of the benefits of the more conservative option using the HIS device. Previous studies have shown this HIS device to be 90% effective in restoring a functional level of knee flexion (> 110° of knee flexion) by the end of treatment which would allow the patient to perform activities of daily living [24, 25]. This previous finding was reinforced by the current study, where the last recorded range of motion (not the knee flexion at the end of treatment) was already at the level required to perform activities of daily living. In both data sources for the 966 subset patents and in the larger dataset of 9842 patients (Datasets 1, 2 and 3), the average last recorded flexion is above the level of flexion required to perform activities of daily living (Navigating stairs—98°; Rising from a low chair—99°; Getting in and out of a car—105°; Tying shoelaces—106°) [27, 28]. For the ROM data taken from physical therapy notes in Dataset 3, more than 90% of patients achieved at least 90° of flexion at the last recorded ROM measurement (93.6%). While data reported in this study is PROM, the difference between AROM and PROM has been reported to be small in previous studies of total knee arthroplasty patients [29–31]. The largest difference between AROM and PROM in these studies was 4%. If that percentage was applied to the 110.7˚ of flexion that was the last recorded ROM for the subset patients, then knee flexion would still be 106.3˚ which would allow the patient to perform all the previously described activities of daily living. There would also be additional gains between the last recorded ROM and the final ROM at the end of treatment which would make it even more likely that the necessary knee flexion would be reached.

While earlier studies exist examining the success of treating knee stiffness with other devices, these are all smaller scale studies [32–35]. To the best of our knowledge there are no similar retrospective large studies investigating the efficacy of other treatment options available for comparison. When comparing HIS therapy to surgical intervention, the reported amount of flexion gained after open surgical release (43.3°) is comparable to gains from the current study and is between the 55.2° average gain made by the patients in the lowest starting ROM (≤ 60°) and the 32.7° average gain in patients with a starting ROM between 61 and 75° [36]. However, the gains from HIS therapy were achieved with none of the risks associated with surgical procedures. Manipulation under anesthesia is only 74% successful in reaching 90° of knee flexion [37]. There is also a critical difference in complications that can occur between the two treatment options. There have been no reported injuries related to the use of Ermi HIS devices in over 25 years of clinical use (FDA Maude database, company complaint logs). Complications after knee manipulation are rare, but can be devastating events, such as tibial plateau fracture, patellar ligament avulsion, pulmonary embolism, and death [21].

Previous studies have noted females are more likely to develop knee arthrofibrosis, and the current study also showed that trend (568 females vs. 398 males) [38, 39]. However, the current study did not find that sex influenced the likelihood of regaining ROM. A potential explanation for this is the HIS device allows each patient to stretch their own selected maximum threshold. This individualized treatment may counteract any sex differences in recovery.

Another benefit of having in home mechanical therapy as an adjunct to physical therapy is that physical therapists are not limited to working on restricted knee motion in the clinic but can also work on strength and neuromuscular re-education in the limited time available. The added value for these select severe motion loss patients is that they make mobility progress both in clinic and at home. This reduces bounce back motion loss between clinic visits which occurs in patients without the added home therapy using an HIS device as an adjunct. This allows the therapist to spend more time on other treatment modalities and contributes to the patient resuming their activities of daily living sooner and returning to work faster.

The fact that there were not any significant differences in initial flexion, last recorded flexion, gain in flexion, or days between delivery and last recorded flexion between workers’ compensation patients and non-workers’ compensation patients is an important finding. In general, workers’ compensation patients demonstrate significantly worse outcomes from treatment when compared to non-workers’ compensation patients [40, 41]. The reason for this is unknown, but could be due to a number of confounding variables, such as psychosocial factors, age, work demands, comorbidities, secondary gain issues, etc. However, none of these confounding variables have been shown to directly correlate with worse outcomes in workers’ compensation patients. The results from this study suggest that the Knee Flexionater is an effective treatment modality for knee arthrofibrosis regardless of workers’ compensation status.

## Limitations

There were several limitations with this study. First, the data was collected from a number of data sources and not via a single defined measurement protocol. The range of motion data for the larger dataset of 9842 patients were taken from prescribing documentation. There was not a record of the exact date of each measurement in the internal database and it was not known whether all measurements were taken with a goniometer. However, data from the subset of patients were collected from physical therapy progress notes, where both the patient's first and last measurements were taken at the same clinic, and were used to validate the data collection methods used for the larger dataset. While differences in median initial measured flexion, last measured flexion and flexion gains were noted between the two collection methods for the subset of 966 patients (5°, 2°,and 3°, respectively), the effect sizes were all considered to be small. As a result of the nature of the study and the large size of the dataset, confounders such as knee surgery type, preoperative knee ROM and duration of physical therapy could not be included. Therefore, future studies should also focus the influence of these factors on regaining ROM using an HIS device.

## Conclusions

High-intensity home mechanical stretch therapy was effective in restoring knee flexion, generally in 2 months or less, and avoiding additional surgery in severe motion loss patients as shown by excellent gains in knee flexion after treatment with the HIS device. This is true regardless of sex, age, or workers’ compensation status. We believe high-intensity stretching should be considered in any patient who is at risk for a secondary motion loss surgery, because in over 90% of these patients, the complications and costs associated with surgery can be avoided.

## Data Availability

The datasets analyzed during the current study are not publicly available due to their ownership by a private company but are available from the corresponding author on reasonable request.

## Abbreviations

AROM: Active range of motion measurement
HIS: High-intensity stretch
MUA: Manipulation under anesthesia
PROM: Passive range of motion measurement
ROM: Range of motion
TERT: Total end-range time
